# Identifying repeat domains in large genomes

**DOI:** 10.1186/gb-2006-7-1-r7

**Published:** 2006-01-31

**Authors:** Degui Zhi, Benjamin J Raphael, Alkes L Price, Haixu Tang, Pavel A Pevzner

**Affiliations:** 1 Bioinformatics Program, University of California, San Diego, CA 92093-0419, USA; 2 Department of Computer Science and Engineering, University of California, San Diego, CA 92093-0114, USA; 3 Department of Genetics, Harvard Medical School, Boston, MA 02115, USA; 4 School of Informatics and Center for Genomics and Bioinformatics, Indiana University, Bloomington, IN 47408, USA

## Abstract

We present a graph-based method for the analysis of repeat families in a repeat library. We build a repeat domain graph that decomposes a repeat library into repeat domains, short subsequences shared by multiple repeat families, and reveals the mosaic structure of repeat families. Our method recovers documented mosaic repeat structures and suggests additional putative ones. Our method is useful for elucidating the evolutionary history of repeats and annotating *de novo *generated repeat libraries.

## Abstract 2

A graph-based method for the analysis of repeat families in a repeat library is presented that helps elucidating the evolutionary history of repeats.

## Background

Repetitive elements form a major fraction of eukaryotic genomes. Though once dismissed as mere junk DNA, they are now recognized as "drivers of genome evolution" [1] whose evolutionary role can be "symbiotic (rather than parasitic)" [2]. Examples of potentially beneficial evolutionary events in which repetitive elements have been implicated include genome rearrangements [1], gene-rich segmental duplications [3], random drift to new biological function [4,5] and increased rate of evolution during times of stress [6,7]. For these and other reasons, the study of repeat elements and their evolution is now emerging as a key area in evolutionary biology.

Individual repeat elements can be grouped into repeat families, each defined by the consensus sequence of its diverged copies. Repeat family libraries, such as Repbase Update libraries [8,9] and RepeatMasker libraries [10], contain consensus sequences of known repeat families. Repeat families often contain shared subsequences, which we call repeat domains. Repeat domains can occur more than once within the same repeat family; for example, the ubiquitous human *Alu *family is dimeric [11]. There are a number of cases of repeat families whose repeat domains are known to have different biological origins, for example, from repeat families with different modes of replication or from distinct retrovirus families. These repeat families and the domains they share are worthy of special attention, since they are assumed to result from interesting evolutionary events. We define a repeat family to be a composite repeat if it contains at least two repeat domains of different biological origin. Of course, discerning the biological origin of a repeat domain is a challenging endeavor. Nevertheless, human Repbase Update documents more than 10 repeat families as composite repeats, including the RICKSHA and Harlequin families. Many other composite repeats contain fragments from different retroviruses. Since composite repeats that contain only fragments of retroviral origin are probably products of retroviral recombinations, these are documented in Repbase Update as retroviral recombinations (see [12] for a review). Composite repeats are likely more than a mere curiosity: one composite repeat, SVA, is the third most active retrotransposon since the human/chimpanzee speciation [13]. An additional example is found in the eel where a composite SINE repeat family borrowed a repeat domain from a different LINE family; this borrowed domain was experimentally shown to greatly enhance the retrotransposition rate of the SINE family [14].

Shared repeat domains yield important insights into repeat evolution, in the same way that multidomain protein organization yields insights into protein evolution [15,16]. However, while the study of protein domains is a well-established research area, the study of repeat domains is still in its infancy. Indeed, RepeatGluer [17] is the only existing algorithm for repeat domain analysis. While RepeatGluer shows promise as a tool for repeat domain analysis, it is computationally intractable for large genomes. For large genomes, we propose that instead of identifying repeat domains *de novo *from genomic sequence, we identify repeat domains by analyzing repeat family libraries that are obtained via other means.

**Figure 1:**
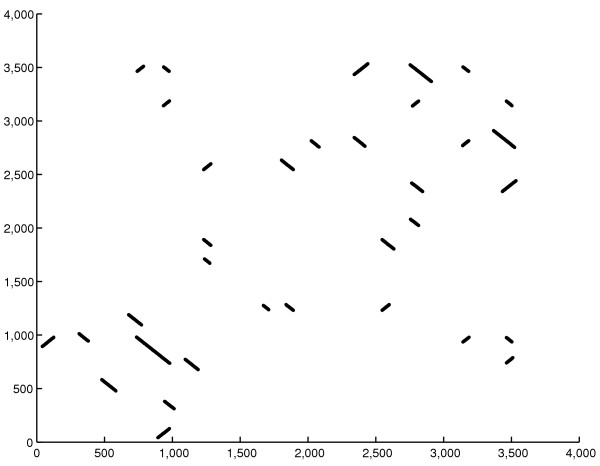
Dot plot of 11 concatenated repeat family sequences from *C. elegans *and *C. briggsae *shows the presence of shared repeat domains. Our repeat domain graph of the same set of sequences is shown in Figure 5.

The main challenge in the analysis of repeat domains is that repeat family consensus sequences typically form a complex mosaic of shared subsequences. This mosaic structure is reminiscent of the mosaic structure of segmental duplications in mammalian genomes [18] (H Tang, Z Jiang, EE Eichler, submitted). Standard sequence comparison tools are unable to capture mosaic structure. These tools reveal local similarities between different repeat families, but do not reveal the structure of shared repeat domains between different families. For example, although a dot plot of the sequences of the 11 *Caenorhabditis elegans *and *C. briggsae *repeat families sharing repeat domains (Figure 1) contains essentially all the information about these repeat families, it is not well-organized and leaves one puzzled about what the repeat domains are. Thus, identifying repeat domains is an important and unsolved problem.

**Figure 2:**
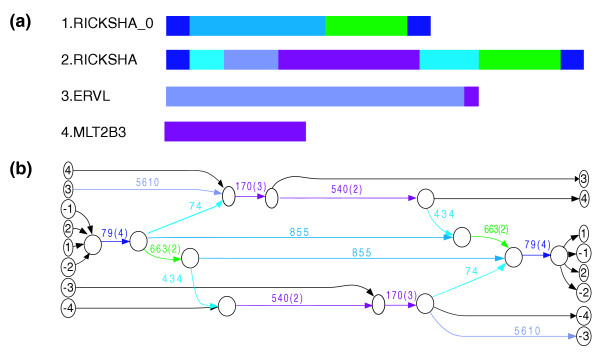
Repeat domain structure and repeat domain graph. **(a) **Diagram of repeat domains shared between RICKSHA and other repeat families. RICKSHA and RICKSHA_0 have 79 bp inverted terminal repeats. In addition, RICKSHA shares some sequences from retroviral elements ERVL and MLT2B. **(b) **Repeat domain graph of the same set of sequences. Each sequence is represented by a path from a source to a sink vertex, where source and sinks are labeled with the ID number in (a). Negative signs refer to the reverse complement sequences (see Results section). Similar parts between sequences are glued into shared edges. Edge label: the number inside the parentheses is the multiplicity and the number outside the parentheses is the length, multiplicity one is omitted.

In this paper, we propose a new framework for analyzing a library of repeat families to identify the mosaic structure of its shared repeat domains. Our main idea is to represent a repeat library by a repeat domain graph that reveals all repeat domains as edges (lines linking between nodes) of the graph, and indicates the order(s) in which those domains appear in the corresponding repeat famili(es). For example, Figure 2 illustrates the domain structure of a selected subset of repeat families sharing repeat domains with the RICKSHA family, and the corresponding repeat domain graph. We describe a method to construct the repeat domain graph from a set of repeat sequences, and we demonstrate methods for analyzing the topology of the repeat domain graph that lead to hypotheses about repeat biology. We apply our method to single-species analyses of human and *C. elegans *repeat family libraries. Our method recovers documented composite repeats in Repbase Update [8,9] and suggests a number of additional putative shared repeat domains in human and *C. elegans*. In addition, we use our method to perform a cross-species comparative analysis of *C. elegans *and *C. briggsae *repeat libraries, and we find a putative ancient repeat domain shared between *C. elegans *and *C. briggsae*. We also demonstrate the application of our method in assisting annotation of repeat libraries that are generated *de novo *from genomic sequence. As numerous new genomes are sequenced and repeat family libraries are automatically constructed, the applications of our method will multiply.

## Results and discussion

Finding all repeat domains is a difficult problem, since repeat family consensus sequences can share subsequences with themselves (for example, *Alu *repeats) or with other repeat families (for example, the composite repeat families described above). Thus, we argue that the ideal method for comparing repeat families and identifying repeat domains should allow for both self-similarities and shared similarities that appear in different orders in different sequences. Such similarities are difficult to capture in traditional multiple alignments that either attempt to align sequences over their entire length (for example, global alignment) or show small conserved regions of similarity (for example, local alignment) with no information about the location of these regions in the original sequence. Recently, several software programs including the Partial Order Alignment (POA) program [19], Threaded Blockset Aligner (TBA) [20], and the A-Bruijn Aligner (ABA) [21] were developed to address these shortcomings. ABA seems particularly well-suited both to the alignment of repeat family consensus sequences and to the decomposition of a library of such consensus sequences into repeat domains since ABA was designed for the alignment of sequences with both repeated and shuffled segments. However, we found that ABA could not automatically generate a repeat domain graph from a repeat library because repeat libraries frequently contain a large number of diverged sequences, including palindromic sequences. Below we describe how to overcome these difficulties.

In addition, since a repeat library typically contains several hundred to several thousand sequences, and the annotation of repeats is typically incomplete, the analysis of a repeat domain graph is a nontrivial task. Below we show several examples illustrating how particular queries in the repeat domain graph can provide powerful systematic analysis of repeat families in a repeat library, how topology of the repeat domain graph can help in elucidating evolutionary history, and how to deal with contaminants, which are common in *de novo *generated repeat libraries.

### Applying the A-Bruijn graph to repeat library analysis: methodology and new algorithms

We represent an alignment of sequences in a repeat library as a directed graph called the repeat domain graph. The repeat domain graph of *n *sequences contains 2*n *source vertices and 2*n *sink vertices. A directed path in the graph from a source to sink vertex represents a sequence or the reverse complement of a sequence in the repeat library. The repeat domain graph typically contains several connected components. Each component corresponds to groups of repeat families with shared repeat domains, and can be analyzed individually. Edges in the repeat domain graph with multiplicity greater than one represent repeat domains that are shared between different repeat families, while single-multiplicity edges correspond to domains unique to a single family.

We construct repeat domain graphs using the framework of A-Bruijn graphs, which were first introduced and applied to the problems of DNA fragment assembly and *de novo *repeat classification in [17], and later extended to the alignment of protein sequences and genomic DNA sequences [21]. The A-Bruijn graph is a general framework for handling sequences with repeated or shuffled domains and is constructed from a set of sequences and a set of pairwise alignments between these sequences. In practice, the A-Bruijn graph of a set of pairwise alignments often contains numerous short cycles, due to inconsistencies among the input alignments. These short cycles obfuscate the identification of the shared domains among these sequences and thus a series of graph heuristics is used for removing short cycles due to inconsistent alignments while retaining longer cycles due to shared domains. We discovered that these approaches were not sufficient to handle two complications that arise in repeat library analysis: namely, the need to align a large number of diverged sequences and the existence of palindromic sequences. The shortcomings of the method were not anticipated or addressed in earlier work because these issues did not arise in the problems addressed there: namely fragment assembly [17], where the input is a large number of very similar (greater than 95%) DNA sequences (reads), and the problems of multiple sequence alignment of a relatively small number of protein sequences or genomic DNA sequences [21]. We developed new algorithms for the construction of the repeat domain graph that are modifications of the methods used to construct and simplify the A-Bruijn graph. Our new algorithms show significant improvement over the existing methods in the handling of inconsistent pairwise alignments and palindromic sequences, both of which are common in repeat libraries constructed *de novo *from genome sequences. These new algorithms are described and compared to the existing methods in the Materials and methods section.

### Analysis of repeat domains in human Repbase

We first built a repeat domain graph of the Repbase library [8,9] of human repeat sequences - the most well annotated repeat library available - in order to test the ability of our method to reveal shared repeat domains and the structure of composite repeats. The resulting repeat domain graph of the 620 sequences in Repbase update contains 9,774 edges and has a complicated topology with 410 connected components, 168 of them containing shared repeat domains (see Additional data file 1 for the entire repeat domain graph, and a list of repeat families contained in each connected component). The largest connected component contains sequences in the library corresponding to the L1 retrotransposon, including the consensus sequences of different families, subfamilies, and partial copies of L1 present in Repbase. Many repeat domains identified in the graph are domains shared by such derivative sequences of a single repeat type. However, other repeat domains are shared sequences between repeat elements of different biological origin. We found 624 such domains by choosing edges in the graph that have minimal length 20, multiplicity greater than 1 and contain sequences whose Repbase annotations suggest different biological origin of the sequences. As there is no ontology of repeat families, we identify 'different biological origin' with a very loose definition: by the first two characters of the repeat family name. We also identify for each repeat family, the length of shared domains and the fraction of its total length containing repeat domains of different biological origin. Table 1 lists the repeat families with the greatest length of such shared domains (Additional data file 2 contains the full list). Near the top of the list are Harlequin and PABL_AI, two repeat families documented in Repbase Update as products of retroviral recombinations. In addition, there are a large number of repeat families with prefixes MER- and HER-, consistent with the observation that retroviral recombination is a dominant feature among repeat families in large mammalian genomes [12]. We remark that the number of 624 repeat domains shared across repeat families is much higher than what is documented in Repbase, suggesting that composite repeats are a rather common phenomenon. However, this conclusion is tempered by the simple criterion that we used to determine biological origin.

**Table 1:** Fifteen repeat families containing domains shared with repeat families of different biological origin

Repeat family	Number of domains	Length of domains	Percent shared
HARLEQUIN	34	6,245	0.9
MER52AI	32	5,375	0.76
HERVL	4	5,117	0.9
ERVL	4	5,117	0.89
HUERS-P3	31	5,106	0.57
LOR1I	54	4,034	0.5
HUERS-P3B	67	3,791	0.51
HERVE	9	3,463	0.44
HERVG25	50	3,431	0.49
HERV35I	54	2,933	0.42
MER51I	45	2,914	0.37
MER4I	48	2,896	0.45
HERVIP10FH	25	2,782	0.54
PABL_AI	52	2,665	0.53

The list is sorted by the total length of shared domains. See Additional data file 4 for the complete table.

In addition to repeat domains, the repeat domain graph also reveals known composite repeats in Repbase.

**Figure 3:**
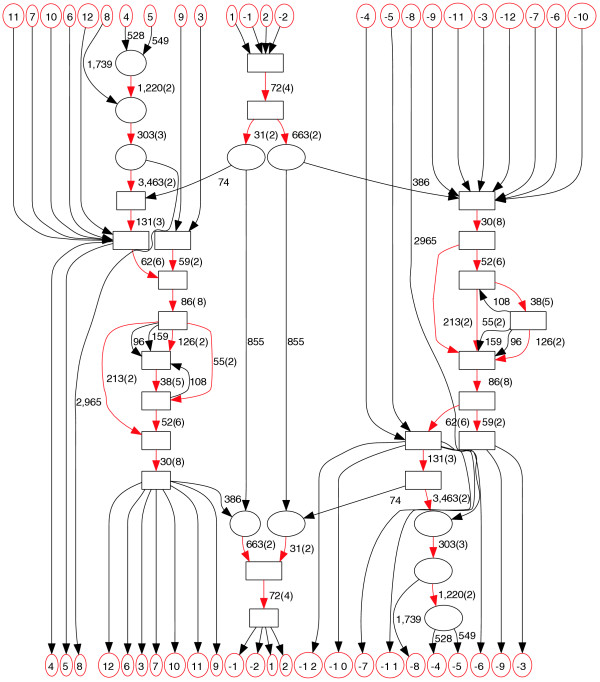
A connected component in the repeat domain graph of the human Repbase. Labeling follows Figure 2b. Edges with multiplicity more than one are highlighted in red. Source/sink labels: 1 = RICKSHA; 2 = RICKSHA_0; 3-12 = various retroviral repeats, including subfamilies of *MLT2 *and the sequences containing the internal part of the endogenous retroviral element *HERVL*.

Figure 3 shows one connected component in the repeat domain graph containing the families RICKSHA, RICKSHA_0, a number of subfamilies of *MLT2 *and the sequences containing the internal part of the endogenous retroviral element *HERVL*. Repbase annotates RICKSHA as a composite repeat that contains 79 base pair (bp) terminal inverted repeats and a 3'-portion of *HERVL *endogenous retrovirus including *MLT2B*, its long terminal repeat (Figure 3). It is believed that RICKSHA replicated before it obtained the retroviral component, and the Repbase entry RICKSHA_0 contains the terminal inverted repeats and different internal sequence from RICKSHA. The repeat domain graph contains two basic paths: the path in the middle containing the edge of length 855, and the path on the left (or right, since they are reverse complement of each other) containing a sequence of red edges. The path in the middle corresponds to the RICKSHA_0 element. The path on the left corresponds to the retroviral elements represented by *MLT2 *and *ERVL*. Interestingly, the path of RICKSHA (sequence number 304) starts and ends in the middle path (the edge of length 72 corresponds to the inverted terminal repeats), but jumps to the path on the left traversing the edges of lengths 74 and 386. This graph vividly illustrates the sequence structure and putative evolutionary history of the RICKSHA element.

We remark that the subtle structure of shared repeat domains in this example are not clearly revealed by traditional row-column multiple alignment programs such as CLUSTALW [22], which align all sequences over their entire lengths. The repeat domain graph removes the restriction of aligning sequences over their entire length, and strikingly reveals the mosaic structure of these repeat families. We further remark that the correspondence between edges and repeat domains is only approximate. Determining the exact boundaries of repeat domains is a challenging problem, similar to the difficulty in defining the boundaries of protein domains. The ambiguity in boundary definition is manifested by complicated structures of short edges in the repeat domain graph. We ameliorate this ambiguity by contracting very short edges (length less than 20).

### Discovering new composite repeats: repeats in *C. elegans*

We built the repeat domain graph of *C. elegans *repeat family library generated by Stein *et al*. [23] with the RECON program [24]. This library contains 377 sequences of total length 251,168 bp. The resulting repeat domain graph contains 2,725 edges that organized into 464 connected components. Of these, 300 components represent 150 repeat families (and their reverse complements) that have neither self-similarities nor similarities with other repeat families. Another 109 connected components represent self-similarities among 86 repeat families that share no similarities with any other families. The remaining 55 connected components reveal the similarities and complex evolutionary relationship between the remaining 142 repeat families.

**Figure 4:**
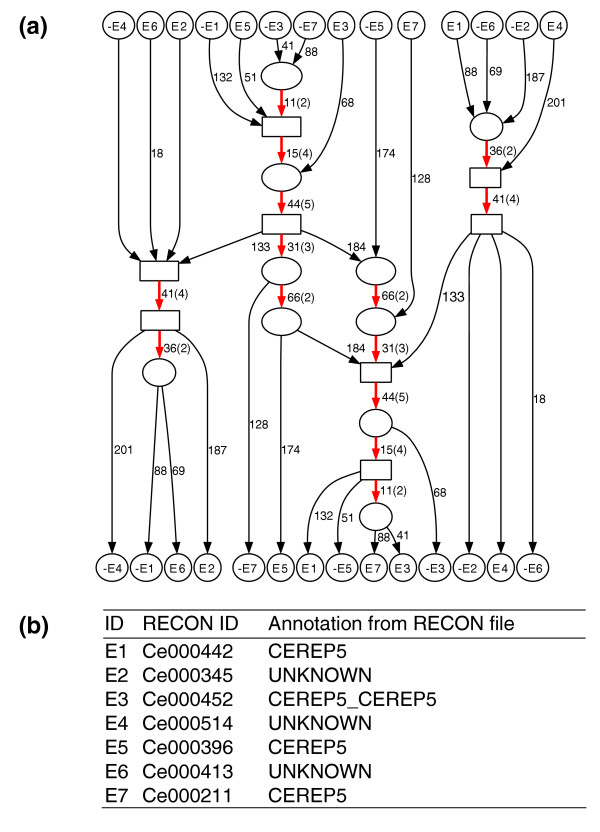
A connected component in the *C. elegans *repeat domain graph. **(a) **Graph topology reveals similarities between seven different repeat families. High-multiplicity edges are colored red. We contract connected subgraphs consisting of edges with a length shorter than 10 (except for edges linked to a source or sink) into boxes to simplify the overall topology of the graph. **(b) **Annotation of the seven families obtained from [23].

We examined one of these 55 connected components that is formed by 7 repeat families (Figure 4). We make the following observations:

1. The complex evolutionary history of these repeat families is reflected in the mosaic structure of repeat domains. For example, repeat family E6 (path from source E6 to sink E6 in Figure 4) is decomposed into five repeat domains. Among them, the repeat domain with length 41 is shared with three other repeat families: E1, E2, and E4.

2. The edges with multiplicity greater than one form two paths - plus the two reflections of the paths resulting from the symmetry of repeat domain graph (red in Figure 4). We refer to these paths as the long path (containing five red edges) and the short path (containing three red edges). These paths delineate important parts of the repeats and may correspond to domains important for the propagation of the repeat elements [23].

3. These repeat families contain different combinations of edges on two red paths in Figure 4. These structures illustrate how one repeat family may borrow repeat domains from another repeat family. Repeat families E6, E2, and E4 contain only the short path; repeat families E5 and E7 contain only the long path; repeat family E3 contains two (partial) copies of the long path, with opposite strand configurations. Interestingly, repeat family E1 contains both paths: part of the long path followed by the short path.

These observations provide a more detailed description of the relationships among repeat families than the simple annotations that are part of the RECON library (Figure 4b). Specifically, the graph reveals a complicated relationship between these repeat families and suggests putative annotations of still unannotated repeat families in the RECON library (for example, those in Figure 4b).

### Comparative repeat domain graph analysis

Comparing repeats across different species is a non-trivial task. Zhang and Wessler [25] compared the transposable elements (TEs) in *Arabidopsis thaliana *and *Brassica oleracea *via TBLASTN searches of the most conserved coding regions for each type of TE. They found nearly all TE lineages are shared between the two plants. Without complete repeat libraries, they were unable to compare repeats on the repeat family level. Stein *et al*. [23] performed a similar study, comparing repeat family libraries from *C. elegans *and *C. briggsae*, and report that "...despite their general similarities, we were not able to systematically identify ortholog pairs among the *C. briggsae *and *C. elegans *repeats ... we found no simple one-to-one mapping between them".

We compare two repeat family libraries by building a comparative repeat domain graph in the following way. Given two libraries X and Y, we first pool the sequences from both libraries into a single union library, then construct the repeat domain graph of the union library, and color the edges in the repeat domain graph according to whether they are from X only, from Y only, or from both X and Y. We call the resulting edge-colored graph the comparative repeat domain graph. Note that alternatively one could construct separate repeat domain graphs for X and Y then compare the two graphs, but this approach would introduce additional complexity in comparing graphs and should give essentially the same results. We further analyze repeat domains shared by both libraries (ancient domains), and repeat domains present in a single sequence (young domains), and study the evolutionary relationship between them.

We formed the comparative repeat domain graph using the *C. elegans *and *C. briggsae *repeat family libraries generated by Stein *et al*. [23] using the RECON algorithm [24]. Indeed, because *C. elegans *and *C. briggsae *diverged roughly 100 million years ago, it is not surprising that only certain repeat domains present in a common ancestor are still present in both species. We are particularly interested in the discovery of these shared ancient repeat domains, whose conservation is suggestive of a role in repeat propagation, or alternatively may be due to horizontal transfer.

The *C. elegans *library contains 377 sequences (with an average length of 666 bp) and the *C. briggsae *library contains 466 sequences (with an average length of 520 bp). We generated pairwise alignments between these 843 sequences and constructed the comparative repeat domain graph. We annotated each edge in the graph as '*C. briggsae *(only)', '*C. elegans *(only)', or 'both'. Our comparison reveals that only 1,810 bp are shared between the two repeat family libraries. These 1,810 bp form nine edges in the comparative repeat domain graph, comprising four connected components (Table 2). Each component is a simple path. These edges match to *Mariner*, *CEREP5 *element, and *PALTTAA2*/*PiggyBac *repeat families.

**Table 2:** Four connected components formed by shared repeat domains (edges shared between *C. briggsae *and *C. elegans*)

Number of edges	Length	Multiplicity	Number of *C. elegans *+ *C. briggsae *families	Annotation
1	61	2	1 + 1	Mariner
1	309	2	1 + 1	Mariner
1	34	10	4 + 4	CEREP5
6	71	34	2 + 16	PALTTAA2/PiggyBac

**Figure 5:**
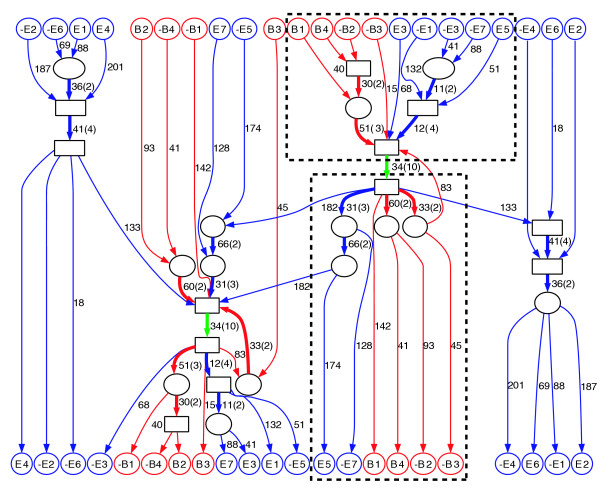
Part of the *C. elegans*/*C. briggsae *comparative repeat domain graph. Labeling follows the legend in Figure 2. Edge color codes: blue, *C. elegans*; red, *C. briggsae*; green, both. Thick edges have multiplicity greater than one. Dashed boxes enclose two subgraphs with a tree topology (see Figure 6 and text).

Length is the total edge length of a component. Multiplicity refers to the highest multiplicity among all edges in a component. The multiplicity may exceed the total number of *C. elegans *and *C. briggsae *families containing the repeat domain, because some repeat families have self-similarities (for example, E3 and B4 each contribute 2 to the multiplicity of the green edge in Figure 5).

We analyzed each of these four connected components. The two shared edges with lengths 61 and 309 are in the same connected component in the comparative repeat domain graph. A translated sequence search revealed that they match essential parts in the transposase-coding sequence of the *Mariner *element. The edge of length 309 matches a set of hypothetical proteins in C. elegans, at residues 117 to 219. Those hypothetical proteins are all closely similar to transposases of other organisms, including Adineta vaga, human, and Stylochus zebra. The edge of length 61 (translated into a 20 amino acid sequence) does not have a significant BLAST result by itself. A BLAST search of the entire repeat family consensus sequence of Cb000007, which contains both edges, gave a result similar to what was obtained by searching the edge of length 309 alone.

**Figure 6:**
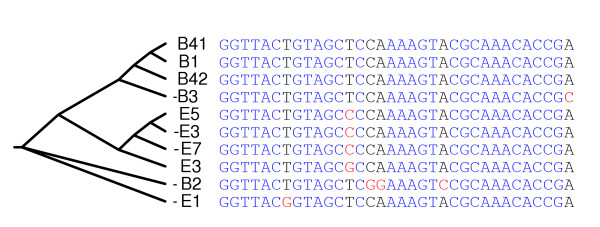
A phylogenetic tree for the sequences that form the shared green edge in Figure 5. Labeling matches that in Figure 5, except that sequence B4 threads through the shared green edge twice, giving two sequences labeled B41 and B42. We remark that the ten sequences show few substitutions; consequently, the topology of this tree is rather reliable despite the fact that the sequences are very short.

Figure 5 shows part of the component of the comparative repeat domain graph containing the edge of length 34 in Table 2. The blue edges correspond to the connected component in the *C. elegans *repeat domain graph shown in Figure 4. The red edges demonstrate four *C. briggsae *repeat families with shared domains. The green edge of length 34 is shared across the two species. We have made a conservative estimate for the statistical significance of the 34 bp edge. Between the five sequences from *C. elegans *and the five sequences from *C. briggsae *(Figure 6b), the closest pair across two species (for example, B1 and E5) has only 1 bp mismatch, for which BLAST reports an E-value (*P* value) of 8E-16. Thus, with the correction of the database size (2.5E5 for *C. elegans *and 2.4E5 for *C. briggsae*), the matching between the two sequences has an E-value of 5E-6.

The comparative repeat domain graph vividly depicts the complex evolutionary history of these repeat families: subtrees split by the green edge (indicated in Figure 5 by dashed boxes) separate repeat families from the two species, and suggest that the repeat domain shared by both species is an ancient repeat domain from a common ancestor, rather than the result of horizontal transfer. Each of these two subtrees induces a phylogeny of the included repeat families. We checked whether these phylogenies were consistent with a phylogeny derived from nucleotide substitutions in the segment of length 34 shared by these sequences (green edge in Figure 5). A phylogenetic tree (Figure 6) of the 10 sequences of length 34 constructed by CLUSTALW gives a phylogenetic tree that is remarkably consistent with the two subtrees in the comparative repeat domain graph. In particular, all three trees group *C. elegans *and *C. briggsae *families together. In addition, sequences -B2 and -B3 share few domains in the trees from the comparative repeat graph, consistent with their long separation on the CLUSTALW tree, while sequences E5 and E7 are close on all three trees. The similarity of the three trees validates the use of the comparative repeat domain graph to infer evolutionary history.

The structure of the comparative repeat domain graph raises a number of interesting and still unresolved evolutionary questions. For example, can we distinguish shared repeat domains between two species that arise from common ancestry from those that arise from horizontal transfer? How have such ancient repeat domains evolved in both genomes, and which repeat domains acquired independently in these genomes have contributed to the evolutionary success of some repeats over the past 100 million years? Finally, we remark that the repeat domain graph shown in Figure 5 was generated from the alignments shown in Figure 1. While Figure 1 contains essentially the same information about local similarities between these repeat families, the graph in Figure 5 organizes this information into a much more interpretable structure.

### Analysis of *de novo *repeat family libraries

We now demonstrate how the repeat domain graph overcomes certain imperfections found in automatically constructed repeat family libraries and directly reveals composite repeats. Repeat family libraries have historically been constructed via manual curation. Recently, algorithms such as RepeatFinder [26], RECON [24], RepeatGluer [17], PILER [27] and RepeatScout [28] are increasingly automating the process of identifying repeat families from genomic sequence. For example, RECON has aided the construction of a library of chicken repeat families [29], and RepeatScout has been used to construct human, mouse and rat repeat family libraries that are nearly as thorough as manually curated libraries. However, the resulting *de novo *libraries (particularly for mammalian genomes) are frequently contaminated by sequences resulting from segmental duplications [18]. We analyzed a human repeat family library that was automatically constructed by RepeatScout, and show how the repeat domain graph helps remove these contaminants and reveals composite repeat families.

We generated a repeat domain graph of a human library generated by RepeatScout containing 1,139 sequences of total length 0.68 M bp. Surprisingly, the resulting graph contains a large connected component that contains more than half of the input sequences. Upon close inspection, we found that this large component is connected by a small number of long edges of single multiplicity. An analysis using BLAT [30] revealed that the instances of each of these long edges in the genome are localized in a small number of narrow genomic regions. This suggests that these long edges do not represent repeat domains, but rather are tandem duplications, a known contaminant of *de novo *repeat identification programs like RECON or RepeatScout.

This discovery revealed an extra benefit of the repeat domain graph for repeat domain analysis: it directly reveals contaminants in automatically generated repeat family libraries. Moreover, the graph suggests a procedure for removing these contaminants. Briefly, we select the longest edge along the path of each repeat family whose total length exceeds 100 bp. We BLAT these edge sequences against the genome sequence and select BLAT hits whose length exceeds 80% of the edge length. We combine BLAT hits into clusters if they are less than 5 Mb apart on the genome. We compute the ratio of the number of hits to the number of clusters, and classify sequences whose ratio exceeds 2 as tandem duplications. Using this approach, 107 repeat families in the RepeatScout library were thus classified as tandem duplications and excluded from further analysis. We remark that this method can detect tandem segmental duplications, but not the dispersed segmental duplications. Distinguishing repeats from dispersed segmental duplications is a challenging and unsolved problem. It is possible that the repeat domain graph might be useful for this problem; however, this is beyond the scope of this paper.

**Figure 7:**
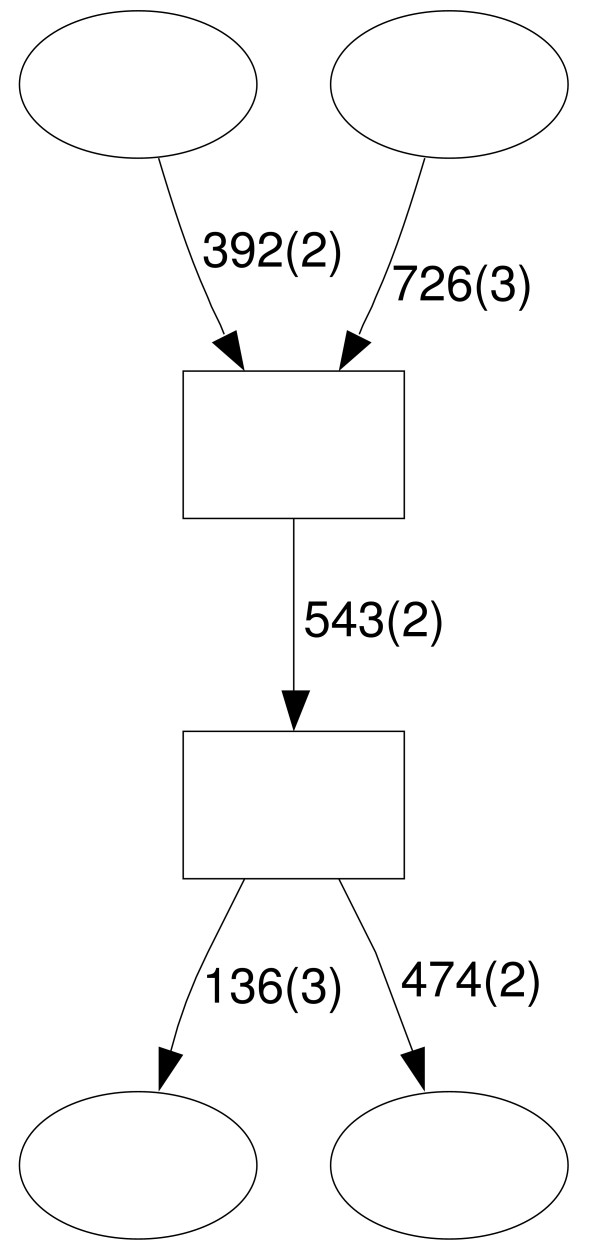
Two Y-forks in a connected component of human RepeatScout library repeat domain graph. The complete graph is available in Additional data file 3.

After removing these contaminants, we built a repeat domain graph from the remaining 992 sequences. The graph contains 885 connected components; the largest component contains 184 sequences. Since we do not have immediate biological annotations for each sequence in the RepeatScout library, we wanted to determine if direct analysis of the repeat domain graph would reveal domain recombinations or composite repeats. A 'signature' in the graph of such an event is a simple branching, or Y-shape fork where two sequences enter a node, and depart on a shared edge. Unfortunately, repeat libraries (including the RepeatScout library) contain a large number of sequences corresponding to partial copies of the same repeat element, which also create Y-shape forks. To reduce the effect of these partial copies, we applied the additional requirement that all three edges in the Y-shape fork should be at least 100 bp long and have multiplicity at least 2. We found six such Y-forks in the repeat domain graph. Furthermore, a single connected component contains three such forks. Closer inspection revealed that two out of the three Y-forks are adjacent (Figure 7) and contain a repeat domain of length 543. We compared the sequences along this edge to human Repbase and found that they correspond to repeat families HERVE, HERVI, and Harlequin. Furthermore, Repbase Update annotates Harlequin as a recombination between several repeat families including HERVE and HERVI. Thus, we were able to directly identify a composite repeat in an unannotated library directly from a signature in the repeat domain graph. The third Y-fork is related to some diverged subfamilies of the MER41 retrovirus. Since the MER41 subfamily has very diverged sequences, accurate subfamily annotation may not be possible. Thus it is difficult to judge whether this Y-fork is due to retroviral recombination or artifacts of alignment programs.

We searched the repeat domain graph from RepeatScout for the RICKSHA composite repeat family described above, but were unable to find it. We determined the reason is that the RepeatScout library itself does not contain RICKSHA, probably due to the high sequence divergence of this repeat family. In addition, we conducted a comparative repeat domain graph analysis (Additional data file 4) for the *de novo *mouse and rat RepeatScout repeat libraries. We found the repeat domain graph helps in purging artifacts in *de novo *repeat libraries, in annotating the library, and in suggesting possible scenarios for repeat family evolution.

## Conclusion

The computational analysis of repeats is becoming increasingly important as additional full genome sequences become available, particularly of repeat rich mammalian and plant genomes. In particular, the problem of identifying shared repeat domains is critical to understanding repeat evolution. This paper describes the first algorithmic advance on automatic identification of repeat domains in large genomes. We have applied our method to single-species analysis of human and *C. elegans *repeat family libraries and cross-species analyses between *C. elegans *and *C. briggsae *libraries and between mouse and rat *de novo *libraries, illustrating the discovery of their mosaic repeat domain structure and revealing interesting clues about repeat evolution.

We have only begun to explore the uses of the repeat domain graph in understanding the relationships between different repeat sequences. We demonstrated that the repeat domain graph reveals known repeat domains of different biological origin. Additional candidates of such domains can be directly identified by signatures in the graph. Repeat families with shared domains that represent putative composite repeat families can be further analyzed to check if their repeat domains do in fact have different biological origins; the PILER algorithm [27], which achieves high specificity in distinguishing between different classes of repeat families, may aid this process. The repeat domain graph opens up additional topics for further research. The library of repeat domains obtained using our decomposition procedure removes all of the redundancy in the original repeat family library. One could build a repeat masking program based on the repeat domain graph and network matching. Repeat subfamily classification algorithms (for example, [31]) can be applied to individual repeat domains to further understand their evolution.

The increasing use of *de novo *repeat identification tools demands careful analysis of the resulting libraries. Our repeat domain graph overcomes certain imperfections found in automatically constructed repeat family libraries, and might prove useful for comparison of repeat libraries generated by different repeat identification tools. As numerous new genomes with high repeat contents, such as mammals and plants, are sequenced and repeat family libraries will be typically automatically constructed, we expect that the applications of our method will multiply.

## Materials and methods

To understand the difficulty in applying the A-Bruijn graph to repeat analysis, we first review the basic concepts of A-Bruijn graph construction, first described in [17]. Briefly, given *n *sequences and a set of pairwise local alignments between them, we first model each sequence *S *= *s*_1_. . .*s*_*k *_as a directed path on *k *vertices. A pairwise local alignment between sequence *S*_*i *_and *S*_*j *_gives the instruction to glue together the paths corresponding to *S*_*i *_and *S*_*j *_at every pair of matched positions in the alignment. The gluing procedure is transitive, that is, if vertex *x *is glued to vertex *y *and vertex *y *is glued to vertex *z*, then vertices *x *and *z *are also glued. Thus, the set of glues define single-linkage clusters of vertices. The A-Bruijn graph construction is completed by contracting all single-linkage clusters of vertices into nodes, and stretching each remaining chains of *l *nodes, each containing *m *vertices, into an edge of length *l *and multiplicity *m*. The resulting A-Bruijn graph can be viewed as an amalgamation of *n *paths: each path corresponds to an input sequence, and similar regions among multiple sequences are represented as edges of high multiplicity. If the inputs are DNA sequences, we take both the direct and reverse strands of each repeat consensus sequence as input. Thus, the A-Bruijn graph of *n *DNA sequences contains 2*n *sources and 2*n *sinks and is symmetric: for any edge representing the alignment of *m *segments, its complement edge in the graph represents the reverse complements of the *m *segments.

In practice, a major obstacle to building an A-Bruijn graph from a set of pairwise alignments is handling inconsistencies in the alignments. These inconsistencies appear as short cycles in the A-Bruijn graph complicating the identification of sequence domains. We define short cycles as cycles of edges with a total length shorter than a predefined parameter, *girth*. We classify short cycles in an A-Bruijn graph as whirls if all edges of the cycle are oriented the same way, or bulges otherwise. The existing method for A-Bruijn graph construction uses an 'apply-all-glues-then-simplify (AAGTS)' strategy. Basically, all glues, that is, pairs of positions that are aligned in one of the input pairwise alignments, are applied to construct an initial A-Bruijn graph (often full of short cycles), and then a series of graph operations are applied that remove bulges and whirls. In [17], for example, the bulge and whirl removal procedure gave an approximate solution to the Maximum Subgraph with Large Girth (MSLG) problem.

**Figure 8:**
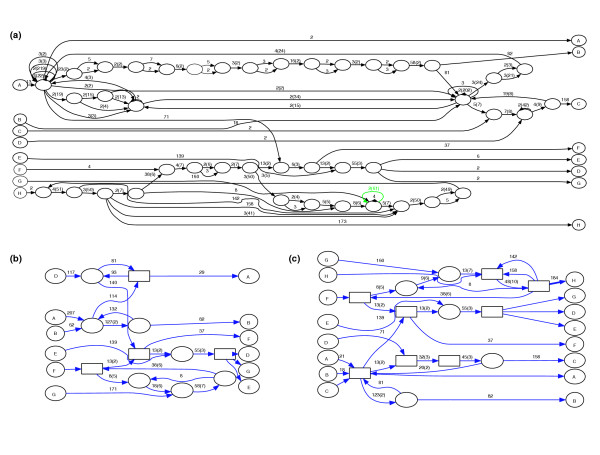
The construction of one connected component in the repeat domain graph of *C. elegans*. **(a) **In the initial A-Bruijn graph, seq. H (Ce000444) and seq. F (Ce000067) are in a same connected component, but many short cycles fragment repeat domains in this graph. **(b) **However, in the graph after the standard bulge and whirl removal procedures (for example, from ABA), due to a whirl removal process starting from the green edge in (a), all glues between seq. H and seq. F are lost and seq. H is in a separate connected component. **(c) **The repeat domain graph constructed with the new whirl handling algorithm and the bulge removal procedure; now seq. H and seq. F are shown to share some significant edges.

When investigating the A-Bruijn approach to the construction of a repeat domain graph, we found the direct application of existing A-Bruijn graph construction algorithms is problematic. The major technical challenge is the internal sequence repeats in repeat consensus sequences. Consensus sequences of repeat families typically contain tandem duplicated subsequences, and directed or inverted terminal repeats. Tandem repeats and directed repeats with repeating unit longer than *girth *are represented as cycles in the repeat domain graph, and those with repeating unit shorter than *girth *are handled by the whirl removal procedure. However, the pairwise alignments between repeat families containing similar repeating units can confound the existing procedure for whirl removal in the A-Bruijn graph. For example, when a tandem repeating unit is duplicated for a modest number *n *times in a repeat, a large number (up to *n*(*n *- 1)/2) of pairwise local alignments can be generated just by self-similarities in this repeat. Even worse, different copies of a tandem duplicated subsequence can have slight variations, which may result in an even larger number of inconsistencies among the set of pairwise local alignments, leading to huge whirl-bulge networks in the A-Bruijn graph. We found the existing whirl removal heuristic is insufficient in handling the complexity in the alignments of repeat consensus sequences in a repeat library. As a result, some similar regions among repeat families are obliterated during bulge/whirl removal and left unglued in the simplified graph. For example, three repeat families, Ce000444, Ce000069 and Ce000167, in the *C. elegans *RECON library [23] contain 2, 3, and 5 copies of some 48 bp long repeat domains. The alignments between these repeat families have extensive pairwise inconsistencies. When applying the existing bulge and whirl removal procedure to simplify the A-Bruijn graph (Figure 8a) for the *C. elegans *repeat library, the resulting graph loses 312 pairs of gluing positions between Ce000444 and Ce000069 and the two repeat families are separated in two different connected components (Figure 8b).

In order to handle such complex inconsistent glues in repeat libraries, we designed and implement a new strategy for filtration of glues. Instead of applying all glues as in the AAGTS approach, we apply the glues one by one and watch for the creation of potential whirls. Specifically, if a pair of positions (*a*, *b*) is about to create a whirl, that is, if position *b *is in a node n(*b*), and n(*b*) contains a position that is on the same sequence as position *a *and is less than *girth *away from position *a*, then the gluing pair (*a*, *b*) is discarded. This conservative gluing procedure prevents the formation of whirls during the A-Bruijn graph construction, and thus a later whirl removal procedure is no longer necessary.

We compare our new whirl-filtration procedure to the existing AAGTS procedure used by ABA by computing the fraction of gluing pairs in the input pairwise alignments that are present in the resulting repeat domain and ABA graphs. To measure the differences between the two methods, we compute the ratio of: the number of gluing pairs in the pairwise alignments that are input to each method; and the number of gluing pairs present in the resulting ABA or repeat domain graph. If there are no inconsistencies in the input alignments, this ratio is 1. If there are inconsistencies in the input alignments, the resulting graphs would have fewer alignment positions, since both methods resolve inconsistencies by removing some aligned positions. Thus, the ratio would be less than 1; however, the best possible ratio is not known. We compute the number of gluing pairs in the input and in the resulting graphs in the following way. Gluing pairs in the input may be redundant. For example, for three positions *i*, *j*, and *k*, if pairs (*i*, *j*), (*j*, *k*) and (*i*, *k*) are aligned in the input, then since pair (*i*, *k*) can be inferred from pairs (*i*, *j*) and (*j*, *k*) by transitivity, only pairs (*i*, *j*) and (*j*, *k*) are sufficient to define same set of gluing operations for constructing the graph. Thus, when we count the number of gluing pairs in the input, we only count nonredundant sets of position pairs; for example, if three positions are aligned transitively, we only count two pairs. In general, if *n *positions are aligned transitively, we only count *n*-1 pairs. To count the number of gluing pairs in the resulting graph, we count the number of positions along the edges with a multiplicity higher than 1. For an edge with multiplicity *m *and length *l*, we count the number of gluing pairs as *l*(*m *- 1). This count is corrected with consideration of over-counting of the positions at common vertices shared by multiple edges.

We find that the whirl-filtration method shows a definite improvement in retaining gluing pairs from the input alignments for the *de novo *derived repeat family libraries C. *elegans *RECON and human RepeatScout (Table 3). Most prominently, for the *C. elegans *RECON library, the repeat domain graph produced by the whirl-filtration procedure retains 97.2% of the input gluing pairs while the graph produced by the AAGTS strategy retains only 89.4%; thus, the new procedure recovers 11,553 gluing pairs. In particular, the alignment between the repeat families Ce000444 and Ce000069 is now correctly represented in the repeat domain graph (Figure 8c,d). Conceptually, the whirl-filtration strategy appears to throw away gluing pairs at the beginning and, therefore, should have produced a graph with fewer glued positions than the previous AAGTS strategy. The explanation for this seeming paradox is the failure of the aggressive whirl removal procedure in the AAGTS approach. In the case of the well-annotated human Repbase, there are fewer inconsistent pairwise alignments in the input, and thus the whirl-filtration and the AAGTS strategies give essentially the same results. Thus, we conclude that the whirl-filtration is more effective when the input alignments contain many inconsistencies, which is often the case for libraries constructed *de novo *from genome sequences.

**Table 3:** Comparison of the AAGTS and whirl-filtration strategies

Repeat Library	Number of non-redundant input POAP	Fraction of input POAP in graph		Difference in POAP
			
		AAGTS	Whirl-filtration	
*C. elegans *RECON	148,989	0.894	0.972	11,553
Human RpeatScout	378,080	0.981	0.994	4,898
Human RepBase	666,100	0.982	0.979	-2,446

We measure the difference between the procedures by the fraction of aligned positions in the input pairwise alignments that are retained in the multiple alignments produced by each graph procedure. POAP, pairs of aligned positions.

The second complication in the construction of the repeat domain graph in repeat is the presence of palindromic sequences. The procedure for constructing an A-Bruijn graph of DNA sequences is designed to preserve the intrinsic symmetric structure of the entire graph. Thus, when gluing a pair of positions, the reverse complement pair of positions is also immediately glued. With palindromic sequences, the order of gluing needs to be coordinated carefully. The existing A-Bruijn construction algorithm did not consider palindromic sequences, and consequently we found that the direct application of the existing procedure often results in a repeat domain graph containing broken paths for a single sequence. We solve this problem by changing the A-Bruijn graph construction procedure so that all glues between positions from same strands are applied before those from opposite strands. Furthermore, we ensure the bulge removal procedure can correctly remove bulges contained in palindromic regions (see Additional data file 5 for details).

While the procedure for construction of the A-Bruin graph is independent of the particular local alignment method, in practice, the resulting repeat domain graph will vary with different input alignments. We determined that cross_match (P Green, unpublished) with the default scoring matrix and the gap penalties used by BLASTN is an effective tool for generating pairwise local alignment of repeat consensus sequences. In comparison, repeat domain graphs produced from alignments with BLASTN (using default parameters) were generally similar but typically contain more edges, thus artificially fragmenting repeat domains.

In all experiments, we selected local alignments with minimal length of 40 and minimal score of 30 (corresponding to BLAST E-value 1E-3) and input these into our method using default parameters with the minimal *girth *(-w) 40. We found that the topology of the repeat domain graph was similar when BLASTN was used to determine the input alignments (data not shown). However, we also observed the importance of filtering out low-complexity alignments, which both cross_match and BLASTN perform under their default options, but using different techniques. When low-complexity filtering was turned off in BLASTN with the '-F F' option, the repeat domain graph generally included larger connected components, reflecting the fact that more low-complexity regions were aligned into repeat domains.

We comment that although our method shares A-Bruijn graph framework with the RepeatGluer program [17], these programs have different goals. RepeatGluer is a *de novo *repeat family identification tool that attempts to identify all repeat families in an input genomic sequence. Unfortunately, it is currently not feasible to run RepeatGluer directly on long genomic sequences. Our approach takes an existing repeat family library as input and decomposes it into repeat domains.

We incorporated our new methods for A-Bruijn graph construction and simplification into a modified version of the ABA program, which is available at the ABA website [32]. The program can also be run online at [33]. Perl scripts used for analyzing the repeat domain graphs are available as Additional data file 6.

## Additional data files

The following additional data are available with the online version of this paper. Additional data file 1 contains a set of browsable HTML files with a complete list of the connected components in the repeat domain graph of human Repbase. Additional data file 2 provides the full version of Table 1, listing repeat families containing domains shared with repeat families of different biological origins. Additional data file 3 is a figure showing a connected component containing three Y-forks in the human RepeatScout library repeat domain graph. Additional data file 4 provides an analysis of the comparative repeat domain graph from mouse and rat RepeatScout repeat libraries. Additional data file 5 shows an example of how palindromic sequences are handled by our revised algorithm. Additional data file 6 provides the repeat domain graph analysis software package

## Supplementary Material

Additional File 1 A zipped file of browsable HTML files with a complete list of the connected components in the repeat domain graph of human Repbase. Click here for file

Additional File 2 The full version of Table 1. The list is sorted by the total length of shared domains. Click here for file

Additional File 3 Labeling follows that of Figure 2b. The three Y-forks are highlighted green. Click here for file

Additional File 4 An analysis of the comparative repeat domain graph from mouse and rat RepeatScout repeat libraries. Click here for file

Additional File 5 An example of how palindromic sequences are handled by our revised algorithm. Click here for file

Additional File 6 Modified ABA program and the perl scripts used for analyzing the repeat domain graphs. Click here for file
