# Supplementary material for: Identifying repeat domains in large genomes
Source: Genome Biol. 2006 Jan 31;7(1):R7. doi: 10.1186/gb-2006-7-1-r7 (PMC1431705; doi:10.1186/gb-2006-7-1-r7)
Supplement: Additional File 1 — A zipped file of browsable HTML files with a complete list of the connected components in the repeat domain graph of human Repbase. [file gb-2006-7-1-r7-S1.gz › html/index.html]

### Connected components of repeat domain graph of human Repbase

  
  
This table includes all connected components (containing more than 1 edge) of the repeat domain graph of human Repbase. The component 6579 contains the RICKSHA repeat family.
  

|  |  |  |
| --- | --- | --- |
| id | num\_edges | num\_repeat\_families |
| 3569 | 6864 | 217 |
| 6579 | 222 | 12 |
| 8688 | 174 | 19 |
| 5589 | 174 | 19 |
| 8099 | 69 | 8 |
| 838 | 69 | 8 |
| 633 | 60 | 6 |
| 8024 | 60 | 6 |
| 5774 | 44 | 10 |
| 8845 | 44 | 10 |
| 7222 | 36 | 4 |
| 5803 | 28 | 7 |
| 8789 | 28 | 7 |
| 8976 | 26 | 4 |
| 6025 | 26 | 4 |
| 890 | 26 | 3 |
| 1049 | 25 | 3 |
| 6002 | 25 | 5 |
| 8944 | 25 | 5 |
| 7268 | 24 | 6 |
| 6841 | 24 | 6 |
| 9334 | 23 | 5 |
| 6718 | 23 | 5 |
| 6381 | 23 | 3 |
| 8328 | 22 | 5 |
| 8072 | 22 | 4 |
| 9435 | 22 | 2 |
| 6807 | 22 | 2 |
| 8255 | 22 | 3 |
| 1176 | 22 | 5 |
| 1096 | 22 | 3 |
| 796 | 22 | 4 |
| 7065 | 21 | 4 |
| 6204 | 21 | 4 |
| 1070 | 20 | 2 |
| 6541 | 19 | 2 |
| 6460 | 19 | 4 |
| 1131 | 19 | 5 |
| 9217 | 19 | 4 |
| 878 | 19 | 5 |
| 1192 | 18 | 4 |
| 6060 | 18 | 4 |
| 8358 | 18 | 4 |
| 6232 | 18 | 4 |
| 9131 | 18 | 4 |
| 5893 | 18 | 4 |
| 5834 | 17 | 4 |
| 6819 | 17 | 4 |
| 7237 | 17 | 4 |
| 8811 | 17 | 4 |
| 6444 | 16 | 4 |
| 6428 | 16 | 4 |
| 6562 | 15 | 3 |
| 7112 | 15 | 3 |
| 9052 | 13 | 3 |
| 9177 | 13 | 3 |
| 867 | 13 | 3 |
| 5869 | 13 | 3 |
| 6695 | 13 | 4 |
| 9317 | 13 | 4 |
| 8870 | 13 | 3 |
| 6127 | 13 | 3 |
| 6397 | 13 | 3 |
| 6105 | 13 | 4 |
| 5899 | 13 | 4 |
| 8118 | 13 | 3 |
| 9241 | 12 | 3 |
| 6508 | 12 | 3 |
| 7002 | 12 | 3 |
| 9596 | 12 | 3 |
| 8047 | 11 | 4 |
| 8106 | 11 | 2 |
| 859 | 11 | 2 |
| 773 | 11 | 4 |
| 9065 | 10 | 3 |
| 6140 | 10 | 3 |
| 8828 | 9 | 3 |
| 5842 | 9 | 3 |
| 9010 | 9 | 3 |
| 5569 | 9 | 3 |
| 6081 | 9 | 3 |
| 8996 | 9 | 3 |
| 6047 | 9 | 3 |
| 6411 | 9 | 3 |
| 9190 | 9 | 3 |
| 8854 | 9 | 3 |
| 5855 | 9 | 3 |
| 8561 | 9 | 3 |
| 9096 | 8 | 2 |
| 6986 | 8 | 2 |
| 7096 | 8 | 2 |
| 6168 | 8 | 2 |
| 6098 | 8 | 2 |
| 9413 | 8 | 2 |
| 9673 | 8 | 2 |
| 8889 | 8 | 2 |
| 6896 | 8 | 2 |
| 9524 | 8 | 2 |
| 6790 | 8 | 2 |
| 6912 | 8 | 2 |
| 5913 | 8 | 2 |
| 6860 | 8 | 1 |
| 9031 | 8 | 2 |
| 9587 | 8 | 2 |
| 9506 | 8 | 2 |
| 9285 | 5 | 2 |
| 779 | 5 | 2 |
| 5905 | 5 | 2 |
| 9610 | 5 | 2 |
| 1076 | 5 | 2 |
| 8881 | 5 | 2 |
| 6683 | 5 | 2 |
| 9363 | 5 | 2 |
| 783 | 5 | 2 |
| 7082 | 5 | 2 |
| 6416 | 5 | 2 |
| 6153 | 5 | 2 |
| 6678 | 5 | 2 |
| 6853 | 5 | 2 |
| 6701 | 5 | 2 |
| 5874 | 5 | 2 |
| 9687 | 5 | 2 |
| 6072 | 5 | 2 |
| 8898 | 5 | 2 |
| 6215 | 5 | 2 |
| 7089 | 5 | 1 |
| 9200 | 5 | 2 |
| 9304 | 5 | 2 |
| 1196 | 5 | 1 |
| 9309 | 5 | 2 |
| 9119 | 5 | 2 |
| 8055 | 5 | 2 |
| 9005 | 5 | 2 |
| 9195 | 5 | 2 |
| 8987 | 5 | 2 |
| 7107 | 5 | 2 |
| 8873 | 5 | 2 |
| 1269 | 5 | 1 |
| 9466 | 5 | 2 |
| 8804 | 5 | 2 |
| 1101 | 5 | 2 |
| 5819 | 5 | 2 |
| 5922 | 5 | 2 |
| 8243 | 5 | 2 |
| 7016 | 5 | 2 |
| 9234 | 5 | 2 |
| 6421 | 5 | 2 |
| 8268 | 5 | 2 |
| 1142 | 5 | 2 |
| 6499 | 5 | 2 |
| 9665 | 5 | 2 |
| 6040 | 5 | 2 |
| 8308 | 5 | 2 |
| 9087 | 5 | 2 |
| 6736 | 5 | 2 |
| 7037 | 5 | 2 |
| 9628 | 5 | 2 |
| 6526 | 5 | 2 |
| 8059 | 5 | 2 |
| 9327 | 5 | 2 |
| 8389 | 4 | 1 |
| 1276 | 4 | 1 |
| 1211 | 4 | 1 |
| 7256 | 4 | 1 |
| 8366 | 4 | 1 |
| 2 | 4 | 1 |
| 8372 | 4 | 1 |
| 1215 | 4 | 1 |
