# Supplementary material for: Identifying repeat domains in large genomes
Source: Genome Biol. 2006 Jan 31;7(1):R7. doi: 10.1186/gb-2006-7-1-r7 (PMC1431705; doi:10.1186/gb-2006-7-1-r7)
Supplement: Additional File 1 — A zipped file of browsable HTML files with a complete list of the connected components in the repeat domain graph of human Repbase. [file gb-2006-7-1-r7-S1.gz › html/subgraphs/1131.html]

|  |  |
| --- | --- |
| id | repbase name |
| 37 | MER2 |
| 45 | MER8 |
| 53 | MER28 |
| 81 | TIGGER2 |
| 553 | MER2B |
