# Supplementary material for: Identifying repeat domains in large genomes
Source: Genome Biol. 2006 Jan 31;7(1):R7. doi: 10.1186/gb-2006-7-1-r7 (PMC1431705; doi:10.1186/gb-2006-7-1-r7)
Supplement: Additional File 1 — A zipped file of browsable HTML files with a complete list of the connected components in the repeat domain graph of human Repbase. [file gb-2006-7-1-r7-S1.gz › html/subgraphs/3569.html]

Graph too big. Here is the dot graph file.

|  |  |
| --- | --- |
| id | repbase name |
| 3 | L1PA2 |
| 4 | L1PA7 |
| 5 | L1PA11 |
| 6 | L1PA15 |
| 7 | L1PB1 |
| 8 | L1PB3 |
| 9 | L1MA2 |
| 10 | L1MA5 |
| 11 | L1MA9 |
| 12 | THE1B |
| 13 | MSTA |
| 14 | MSTC |
| 15 | MLT1A |
| 16 | MLT1B |
| 17 | MLT1C |
| 18 | MLT1D |
| 19 | MLT1E |
| 20 | MLT1F |
| 39 | MER4A |
| 40 | MER4B |
| 41 | MER4C |
| 50 | MER21 |
| 51 | MER21B |
| 57 | MER39 |
| 85 | HERVK |
| 88 | HERVE |
| 90 | HERVI |
| 92 | HERVKC4 |
| 93 | MER4I |
| 94 | MER49 |
| 95 | MER4D |
| 96 | MER39B |
| 99 | HERV3 |
| 100 | HERV9 |
| 104 | L1 |
| 105 | L1MA10 |
| 106 | L1MB3 |
| 107 | L1MB7 |
| 108 | L1MC2 |
| 109 | L1MC3 |
| 110 | L1MC4 |
| 111 | L1MD1 |
| 112 | L1MD2 |
| 113 | L1ME2 |
| 114 | L1ME3A |
| 118 | LTR8 |
| 121 | MER34 |
| 142 | MER72 |
| 151 | MLT1G |
| 154 | MER57I |
| 155 | MER65I |
| 156 | MER41I |
| 158 | L1M2\_5 |
| 173 | HERV17 |
| 188 | HERV23 |
| 198 | MER31I |
| 208 | HERVK9I |
| 209 | HERVH48I |
| 217 | LTR29 |
| 219 | LTR31 |
| 226 | MLT1H |
| 227 | L1P\_MA2 |
| 234 | L1PA12\_5 |
| 235 | L1PA16\_5 |
| 236 | L1PBA\_5 |
| 238 | L1M3A\_5 |
| 239 | L1M3B\_5 |
| 240 | L1M3C\_5 |
| 241 | L1M3D\_5 |
| 242 | L1MB6\_5 |
| 243 | L1MCA\_5 |
| 244 | L1MCB\_5 |
| 245 | L1MEA\_5 |
| 246 | L1MEC\_5 |
| 247 | L1ME\_ORF2 |
| 254 | HARLEQUIN |
| 258 | LTR34 |
| 259 | LTR35 |
| 263 | MER66I |
| 272 | HERVK22I |
| 273 | MER51I |
| 281 | LTR39 |
| 286 | HUERS-P3 |
| 287 | HUERS-P3B |
| 288 | HUERS-P2 |
| 291 | HERVG25 |
| 296 | L1MC5 |
| 298 | HERVFH21 |
| 307 | HERVK3I |
| 312 | LTR48 |
| 313 | LTR48B |
| 314 | LTR49 |
| 315 | LTR8A |
| 319 | LTR51 |
| 329 | LTR54 |
| 333 | MER61I |
| 334 | HERVL68 |
| 343 | LTR59 |
| 344 | MER4BI |
| 345 | MER50I |
| 346 | LOR1I |
| 352 | MLT1E1 |
| 356 | MLT1E2 |
| 358 | MLT1G1 |
| 359 | L1MCC\_5 |
| 361 | HERVK11I |
| 362 | HERVK13I |
| 365 | HERV49I |
| 366 | HERV15I |
| 373 | MLT1F1 |
| 382 | L1ME4 |
| 383 | MLT1H1 |
| 387 | MLT1G2 |
| 388 | MSTA1 |
| 392 | PRIMA41 |
| 396 | L1M3DE\_5 |
| 398 | MER4E |
| 401 | LTR54B |
| 403 | MLT1G3 |
| 404 | MSTA2 |
| 408 | MLT1C1 |
| 409 | MSTD |
| 411 | L1MED\_5 |
| 412 | L1ME5 |
| 416 | MER21A |
| 417 | MER34B |
| 423 | MER83AI |
| 424 | MER83BI |
| 425 | MER84I |
| 427 | L1PA7\_5 |
| 428 | L1PA13\_5 |
| 429 | L1M1\_5 |
| 430 | L1M2A\_5 |
| 431 | L1M1B\_5 |
| 432 | L1MB3\_5 |
| 433 | L1MDB\_5 |
| 434 | L1HS |
| 435 | L1PA3 |
| 436 | L1PA4 |
| 437 | L1PA5 |
| 438 | L1PA6 |
| 439 | L1PA8 |
| 440 | L1PA10 |
| 441 | L1PA12 |
| 442 | L1PA13 |
| 443 | L1PA14 |
| 444 | L1PA16 |
| 445 | L1PB2 |
| 446 | L1PB4 |
| 447 | L1MA1 |
| 448 | L1MA3 |
| 449 | L1MA4 |
| 450 | L1MA4A |
| 451 | L1MA5A |
| 452 | L1MA6 |
| 453 | L1MA7 |
| 454 | L1MA8 |
| 455 | L1MB1 |
| 456 | L1MB2 |
| 457 | L1MB4 |
| 458 | L1MB5 |
| 459 | L1MB8 |
| 460 | L1ME1 |
| 461 | L1ME3 |
| 462 | L1ME4A |
| 463 | L1MC1 |
| 464 | L1MD3 |
| 466 | L1MA9\_5 |
| 467 | L1MDA\_5 |
| 468 | L1MEB\_5 |
| 488 | THE1A |
| 489 | THE1C |
| 490 | MSTB |
| 491 | MSTB1 |
| 501 | HERVK11DI |
| 503 | HERV30I |
| 504 | HERV19I |
| 510 | MER21I |
| 511 | PABL\_AI |
| 512 | PABL\_BI |
| 513 | MER52AI |
| 514 | HERV57I |
| 516 | HERV38I |
| 517 | L1M2B\_5 |
| 518 | L1M2C\_5 |
| 519 | HERVFH19I |
| 541 | MER72B |
| 544 | MER34C |
| 546 | HERVP71A\_I |
| 552 | L1PREC1 |
| 557 | MLT1A1 |
| 558 | L1PREC2 |
| 560 | L1M2A1\_5 |
| 561 | MLT1E1A |
| 562 | MER4E1 |
| 563 | PRIMA4\_I |
| 564 | PRIMA4\_LTR |
| 565 | L1M4B |
| 566 | L1PA14\_5 |
| 568 | HAL1C |
| 569 | HERVIP10F |
| 570 | MLT1F2 |
| 572 | HERVIP10FH |
| 574 | MER34B\_I |
| 575 | MER57A\_I |
| 576 | PRIMAX\_I |
| 582 | MER21C |
| 584 | L1PBA1\_5 |
| 585 | L1MB4\_5 |
| 593 | HERV39 |
| 594 | MLT1H2 |
| 596 | MER4A1 |
| 597 | MER4D1 |
| 598 | THE1D |
| 617 | HERV35I |
| 619 | HERV-K14CI |
| 620 | HERV-K14I |
