# Supplementary material for: Identifying repeat domains in large genomes
Source: Genome Biol. 2006 Jan 31;7(1):R7. doi: 10.1186/gb-2006-7-1-r7 (PMC1431705; doi:10.1186/gb-2006-7-1-r7)
Supplement: Additional File 1 — A zipped file of browsable HTML files with a complete list of the connected components in the repeat domain graph of human Repbase. [file gb-2006-7-1-r7-S1.gz › html/subgraphs/5589.html]

|  |  |
| --- | --- |
| id | repbase name |
| 98 | MER61 |
| 116 | LTR1 |
| 180 | LTR20 |
| 206 | LTR25 |
| 215 | LTR27 |
| 216 | LTR28 |
| 220 | MER61B |
| 221 | MER61C |
| 276 | MER52A |
| 277 | MER52B |
| 278 | MER52C |
| 290 | LTR20B |
| 355 | LTR1B |
| 538 | LTR1C |
| 543 | LTR1D |
| 547 | LTR27B |
| 551 | LTR77 |
| 580 | MER52D |
| 583 | LTR20C |
