# Supplementary material for: Identifying repeat domains in large genomes
Source: Genome Biol. 2006 Jan 31;7(1):R7. doi: 10.1186/gb-2006-7-1-r7 (PMC1431705; doi:10.1186/gb-2006-7-1-r7)
Supplement: Additional File 1 — A zipped file of browsable HTML files with a complete list of the connected components in the repeat domain graph of human Repbase. [file gb-2006-7-1-r7-S1.gz › html/subgraphs/6579.html]

|  |  |
| --- | --- |
| id | repbase name |
| 24 | MLT2A1 |
| 25 | MLT2B2 |
| 26 | MLT2C2 |
| 27 | MLT2D |
| 84 | HERVL |
| 172 | HERV16 |
| 261 | MLT2A2 |
| 304 | RICKSHA |
| 397 | RICKSHA\_0 |
| 421 | ERVL |
| 578 | MLT2B3 |
| 590 | MLT2B4 |
