# Supplementary material for: Identifying repeat domains in large genomes
Source: Genome Biol. 2006 Jan 31;7(1):R7. doi: 10.1186/gb-2006-7-1-r7 (PMC1431705; doi:10.1186/gb-2006-7-1-r7)
Supplement: Additional File 4 — An analysis of the comparative repeat domain graph from mouse and rat RepeatScout repeat libraries. [file gb-2006-7-1-r7-S4.doc]

Comparative repeat domain graph analysis of mouse and rat RepeatScout libraries

While *C. elegans* and *C. briggsae* repeat families have few similarities, mouse and rat repeat families are quite similar, due to the shorter time since speciation. In fact, Repbase Update (Jurka 1998; 2000) contains only a single library of rodent repeat families. This library was largely constructed from manual curation of mouse repeat families. Due to the large size and repeat-rich nature of these and other mammalian genomes, *de novo* construction of repeat libraries from genomic sequence is a daunting algorithmic problem. Only recently were separate repeat libraries for mouse and rat generated using RepeatScout (Price et al. 2005), enabling a comparison of mouse and rat repeat families.

The input mouse library contains 886 sequences of total length 1.2M bp and the input rat library contains 831 sequences of total length 0.5M bp. We generated a comparative repeat domain graph using our graph-based method. The resulting graph contained a large connected component that contains 59% of the input sequences. Upon close inspection, we found that this large component was connected by a small number of long edges of single multiplicity. As in the analysis of human RepeatScout library (see Section 2.5), we determined that these long edges represented tandem duplications and used the same procedure to remove them. 261 mouse entries and 150 rat entries in the RepeatScout are thus classified as tandem duplications and excluded from further analysis.

| genome | seqs. | # edges | avg. edge len | avg. edge mul |
| --- | --- | --- | --- | --- |
| Mouse only | 541 | 1542 | 298.9 | 1.2 |
| Rat only | 596 | 1762 | 256.7 | 1.2 |
| both | 85 | 244 | 148.8 | 6.8 |

Table 1 The average edge length and multiplicity of mouse/rat comparative repeat domain graph constructed from their RepeatScout libraries.

We build the comparative repeat domain graph with the remaining entries by selecting all pairwise alignments between the entries with a score cutoff 30. The graph contains 9446 edges, of which 6598 edges lie in the largest connected component. The largest component contains many edges that match to subparts of the LINE element L1 as well edges that match to some LTRs and SINEs. In order to analyze the long domains in the L1 repeat family, we used a more strict score cutoff of 350 for pairwise alignments, resulting a graph containing smaller number of edges (Table 1).

The largest component (Figure 1) of the resulting mouse/rat comparative repeat domain graph contains 53 sequences, 23 from mouse and 30 from rat. All these entries are related to L1 (with the entire or a significant portion of the sequence aligned to L1).

To identify prominent L1 repeat domains, we weight each edge in the repeat domain graph by the number of times the repeat domain is present in the genome; i.e., we assign a copy number to each edge in the following way. We run RepeatMasker with the RepeatScout libraries against the mouse and rat X chromosome sequences. We create a counter for each edge. For each RepeatMasker hit matching to a sub-region of an entry in the RepeatScout libraries, we identify edges in the repeat domain graph corresponding to the matched sub-region, and increment the counters for these edges.

We observe a number of features in the graph that correspond to known knowledge to L1.

1. The topology of the graph contains only one main path, suggesting that there is mainly one repeat family in this component. Indeed all but two edges in the graph match to subparts of L1.
2. Edge copy numbers on the 3’ end (~13,000) are significantly higher than that of the 5’ end (~5,000), reflecting the fact that there are frequent 5’ end truncations of L1 after insertion.
3. The tree-like structure at the 5’ end has a mouse specific branch and a rat specific branch, reflecting that L1 underwent significant expansions after the speciation.
4. The tree-like structure at the 3’ end split into three major “sub-trees”. One is mouse-specific, one is rat-specific, and one is shared across the two species. The shared “sub-tree” corresponds to the Lx subfamilies, which predate the speciation.
5. There is a set of multi-parallel edges close to the 5’ end, representing a region of high variability. Interestingly, the lengths of these edges differ by some multiple of 3. Indeed this region is inside the ORF1 coding region.
6. There are two major mouse specific branches at the 5’ end, each contains a short directed cycle consisting of edges with high copy numbers. The two branches correspond to the A-type and the F-type of L1Md subfamilies, which are known to contain distinct domains of tandem repeats at their 5’ region that may be serve as alternative promoters (Loeb et al. 1986).
7. There are other directed cycles in this component which could correspond to other interesting features of L1 repeats.

We remark that the graph provides a principled way to extract biological knowledge out of a *de novo* constructed repeat library. During the graph construction we use zero prior knowledge on the repeat content in mouse or rat. The above results demonstrate the potential of application the repeat decomposition graph approach to post-processing *de novo* repeat libraries: purging tandem duplications, family/subfamily classification of *de novo* repeat families, and gaining biological insights.


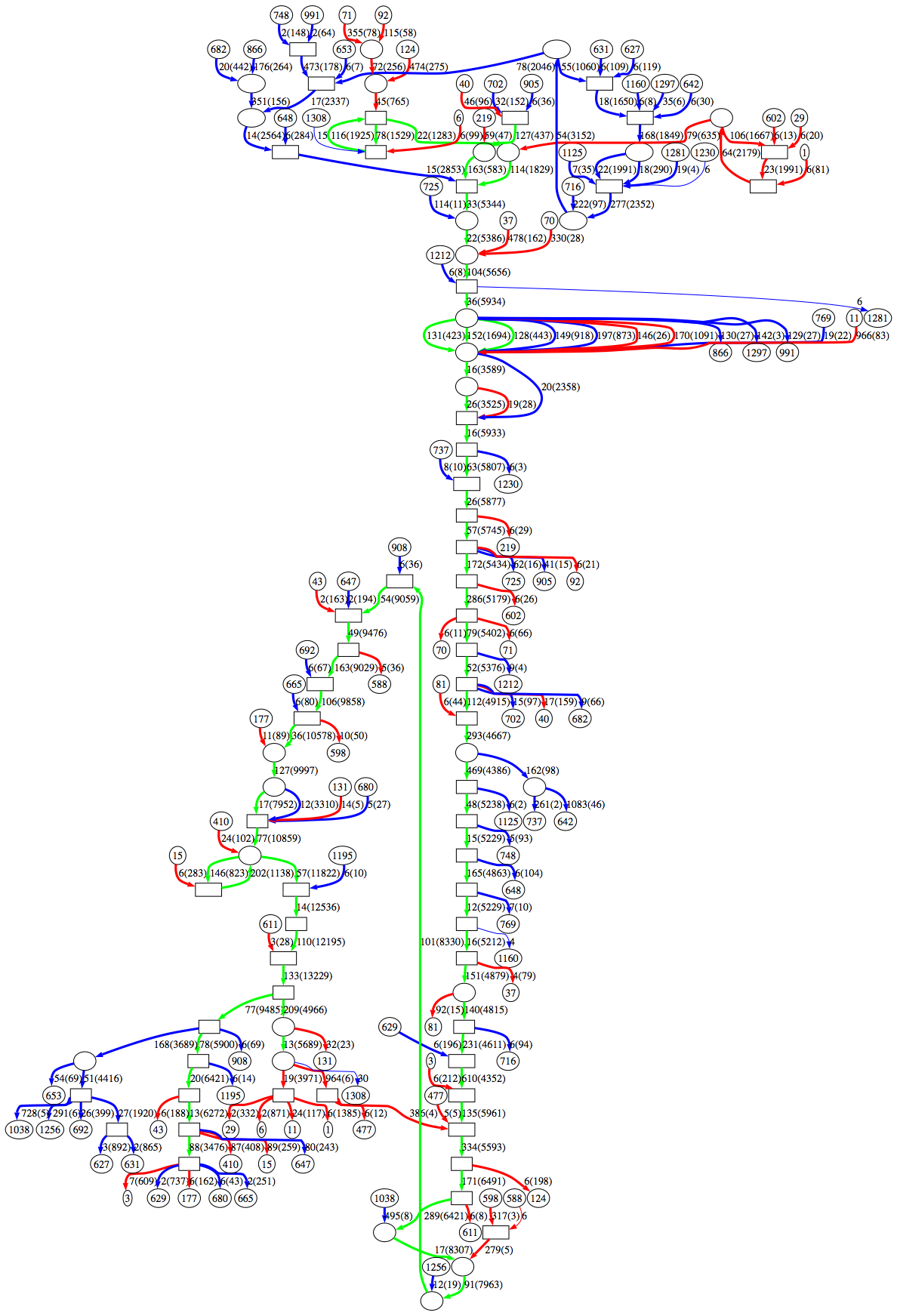


3

2

4

2

5

Figure 1: Largest connected component of mouse/rat comparative repeat domain graph. Each edge is labeled *l*(*c*), where *l* is the length and *c* is the copy number of the sequence in the X chromosomes. Edges with length <10 are contracted. Sources (top of figure) are 5’ ends of L1 sequences, while sinks (bottom) are 3’ ends. Observations in text are highlighted with boxes and arrows with corresponding numbers.

8. Jurka, J. (1998). "Repeats in genomic DNA: mining and meaning." Curr Opin Struct Biol 8(3): 333-7.

9. Jurka, J. (2000). "Repbase update: a database and an electronic journal of repetitive elements." Trends Genet 16(9): 418-20.

28. Price, A., Jones, N. and Pevzner, P. (2005). *De novo* identification of repeat families in large genomes. Proceedings of the 13th Annual International conference on Intelligent Systems for Molecular Biology (ISMB-05), Detroit, Michigan.

34. Loeb, D. D., Padgett, R. W., Hardies, S. C., Shehee, W. R., Comer, M. B., Edgell, M. H. and Hutchison, C. A., 3rd (1986). "The sequence of a large L1Md element reveals a tandemly repeated 5' end and several features found in retrotransposons." Mol Cell Biol 6(1): 168-82.
