# Supplementary material for: Identifying repeat domains in large genomes
Source: Genome Biol. 2006 Jan 31;7(1):R7. doi: 10.1186/gb-2006-7-1-r7 (PMC1431705; doi:10.1186/gb-2006-7-1-r7)
Supplement: Additional File 5 — An example of how palindromic sequences are handled by our revised algorithm. [file gb-2006-7-1-r7-S5.doc]

Here we give an example to illustrate the improvement on handling repeat families with palindromic structure. In the C. briggsae RECON library, the center part of the repeat family Cb000083 (Stein et al 2003 annotated as AT_rich_Low_complexity___TC1_DNA/Tc1) is palindromic. Part of its sequence aligned with the repeat family Cb000214 (Stein et al 2003 annotated as unknown). Without the new procedure for handling such palindromes, the repeat domain graph (Figure 1(a)) breaks at the middle of the palindromic sequence of Cb000083. The new procedure (Figure 1(b)) brings them together to make a complete path.

|  | (a) |
| --- | --- |
|  | (b) |

**Figure 1 Handling self-palindromic sequences in the *C. briggsae* RECON library (as part of the C. elegans/C. briggsae comparative repeat domain graph). Sequences numbered 11 and 192 have corresponding RECON ID’s of Cb000083, and Cb000214. (a) Direct use of ABA breaks the path for sequence 11 (and –11) at the nodes labeled 11/-11. (b) The new graph building procedure correctly contains a single path for sequence 11.**
